# MEK/ERK signaling pathway is required for enterovirus 71 replication in immature dendritic cells

**DOI:** 10.1186/s12985-014-0227-7

**Published:** 2014-12-30

**Authors:** Weifeng Shi, Xueling Hou, Hongjun Peng, Li Zhang, Yuanyuan Li, Zhiwen Gu, Qingbo Jiang, Mei Shi, Yun Ji, Jingting Jiang

**Affiliations:** Department of Clinical Laboratory, The Third Affiliated Hospital of Soochow University, 185 Juqian Street, Changzhou, Jiangsu 213003 PR China; Department of Tumor Biological Treatment, The Third Affiliated Hospital of Soochow University, 185 Juqian Street, Changzhou, 213003 China

**Keywords:** Enterovirus 71, Virus replication, MEK/ERK, SOS1, Immature dendritic cells

## Abstract

**Background:**

The mitogen-activated protein kinase kinase/extracellular-signal-regulated kinase (MEK/ERK) signaling pathway is involved in viral life cycle. However, the effect of MEK/ERK pathway in enterovirus 71(EV71)-infected immature dendritic cells (iDCs) is still unclear.

**Methods:**

Human peripheral blood mononuclear cells (PBMCs) were isolated and induced to generate iDCs. Unifected iDCs and EV71-infected iDCs with a multiplicity of infection (MOI = 5) were analyzed by flow cytometry. Differential gene expressions of MEK/ERK signaling pathway molecules in EV71-infected iDCs were performed by PCR arrays. The phosphorylation of MEK/ERK pathway molecules in EV71-infected iDCs preincubated without or with U0126 (20 μM) at indicated times was detected by Western blot. The concentrations of IL-1α, IL-2, IL-6, IL-12, TNF-α, IFN-α1, IFN-β and IFN-γ in culture supernatant were analyzed by the luminex fluorescent technique.

**Results:**

When iDCs were infected with EV71 for 24 h, the percentage of CD80, CD83, CD86 and HLA-DR expressed on iDCs significantly increased. PCR arrays showed that gene expressions of molecules in MEK/ERK signaling pathway were remarkably upregulated in EV71-infected iDCs. EV71 infection activated both MEK1/2 and ERK1/2, which phosphorylated their downstream transcription factor c-Fos, c-Jun, c-myc and Elk1. Importantly, the treatment of U0126 significantly inhibited MEK/ERK signaling pathway molecules and severely impaired virus replication., Additionally, EV71 infection promoted the expression of son of sevenless (SOS1) and increased the secretion of IL-1α, IL-2, IL-6, IL-12, TNF-α,IFN-β and IFN-γ. Furthermore,the release of IL-1α, IL-2,IL-6 and TNF-α could be effectively suppressed by inhibitor U0126.

**Conclusions:**

Our data suggest that the MEK/ERK signaling pathway plays an important role in EV71-infected iDCs and these molecules may be potential targets for the development of new anti-EV71 drugs.

## Introduction

EV71 is a member of the *Picornaviridae* family, which is composed of a large number of small non-enveloped, positive strand RNA viruses with a genome size of approximately 7500 bp [1,2]. Increasing evidences indicate that EV71 has become the major etiological agent of current outbreaks of hand, foot, and mouth disease (HFMD) in the Asia-Pacific region, including China [3-5]. In addition, the clinical features of EV71 infection vary from mild HFMD or herpangina to aseptic meningitis, encephalitis, pulmonary edema and even death [6-8]. Premature or impaired immunity upon EV71 infection has been associated with increased morbidity and mortality [9,10]. However, the molecular pathogenesis of EV71 infection remains elusive.

Mitogen activated protein kinases (MAPKs) are central molecules mediating signaling pathways in innate immunity. They belong to a family of serine/threonine protein kinases which are largely conserved among eukaryotes and involved in many cellular processes such as inflammation, proliferation, differentiation, movement, and death [11,12]. To date, seven distinct groups of MAPKs have been characterized in mammalian cells, including extracellular regulated kinases (ERK1/2), c-Jun N-terminal kinases (JNK1/2/3), p38 MAPK (p38 α/β/γ/δ), ERK3/4, ERK5, ERK7/8 and Nemo-like kinase (NLK) [13,14]. Among these, the most extensively studied are ERK1/2, JNKs and p38 MAPKs [15,16]. Acute activation of MAPK signaling cascades is utilized by both viruses for their replication and hosts for defense against the viruses [17,18]. Virus infection activates the MEK/ERK signaling pathway, which further promotes activation of transcription factors and increases the secretion of different cytokines to affect virus propagation [19,20]. Furthermore, transcription of many MAPK-regulated pro-inflammatory or antiviral cytokines such as IL-2, IL-6, IFN-β and TNF-α is dependent on transcription factor NF-κB, c-Fos and c-Jun.

DCs are the first line of host defense. They initiate specific host immune responses by capturing, processing, and presenting antigens through MHC-I and MHC-II molecules on the cellular surface and activating naïve T cells [21,22]. The effect of MEK/ERK signaling cascades during EV71 infection in many other cells has been extensively explored. For example, it has been reported that the MEK/ERK signal cascade is required for replication of EV71 in embryonic rhabdomyosarcoma (RD) cells and HEK cells [23,24]. However, MEK/ERK signaling pathway in EV71-infected iDCs remains largely unknown.

To explore the roles of MEK/ERK signaling pathway in EV71-infected iDCs, we induced iDCs from PBMCs in the presence of granulocyte-macrophage colony-stimulating factor (GM-CSF) and IL-4. The iDCs were then infected with EV71 and used to investigate the effect and mechanism of MEK/ERK signaling pathway associated molecules during EV71 infection.

## Results

### EV71 infection increased activation of iDCs

**Figure 1:**
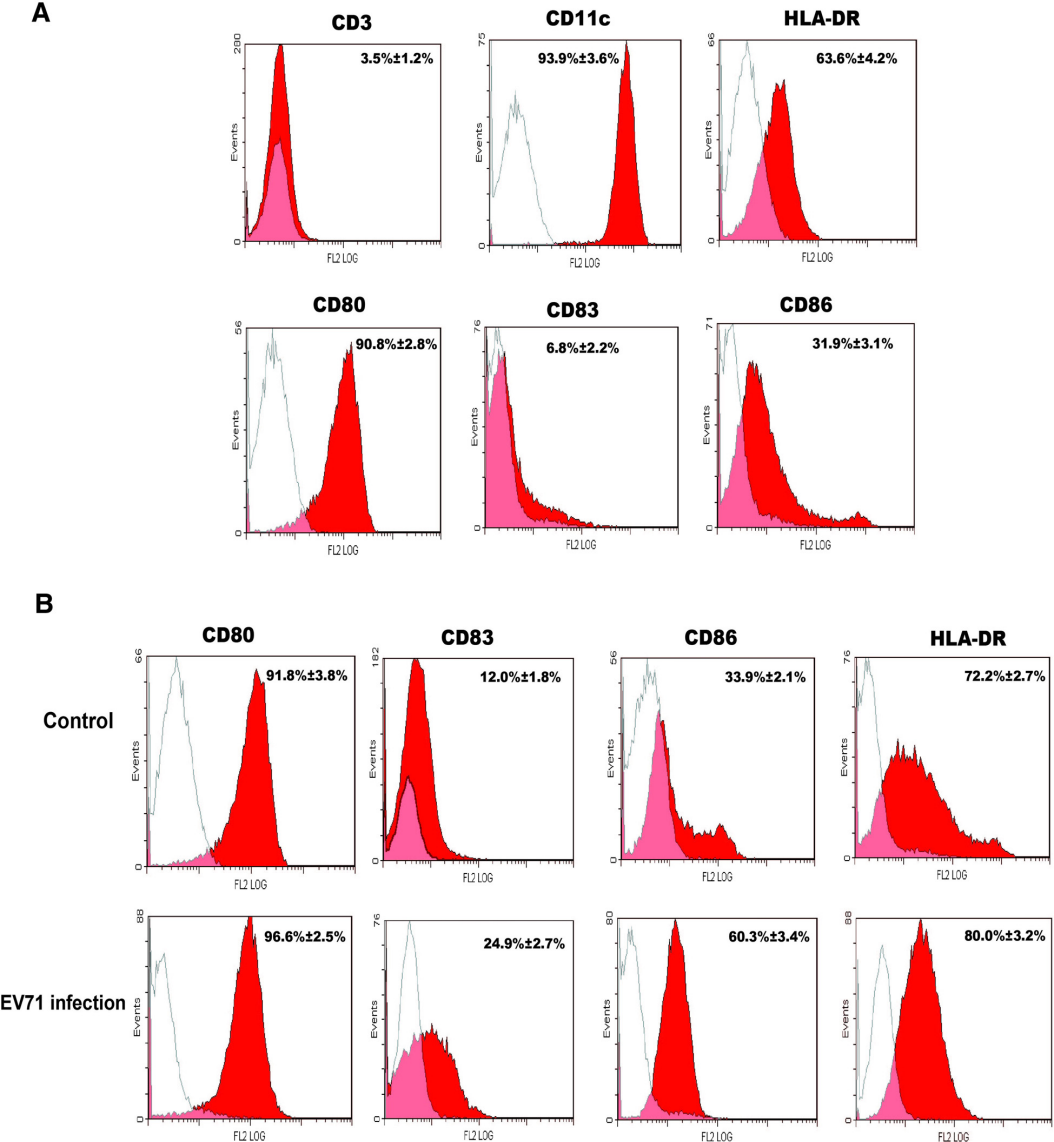
**Flow cytometric analysis of human blood monocyte-derived iDCs and EV71-infected iDCs. (A)** Monocytes cultured in the medium containing GM-CSF and IL-4 for 7 days were analyzed by flow cytometry with isotype-matched control (thin lines) using specific antibodies (red lines). The data shown are representative of three independent experiments. **(B)** Uninfected iDCs (control) or iDCs infected with EV71 (MOI = 5) were analyzed using flow cytometry for the expression of CD80, CD83, CD86 and HLA-DR at 24 h p.i. using isotype-matched control (thin lines) and specific antibodies (red lines). The data were expressed as mean ± SE from three independent experiments and analyzed by Chi-square test.

iDCs were prepared from monocytes purified from peripheral blood by induction with GM-CSF and IL-4. Flow cytometric analysis indicated that most iDCs were positive for CD11c, HLA-DR and CD80, but only 3.5% ± 1.2% and 6.8% ± 2.2% of cells were positive for CD3 and CD83, respectively, confirming that they were indeed iDCs (Figure 1A). After being infected with EV71 (MOI = 5) for 24 h, the percentages of CD80 and HLA-DR didn’t increase in EV71-infected iDCs compared to non-infected iDCs (*p* > 0.05). However, CD83 and CD86 distinctly increased at 24 h postinfection (p.i.) (*p* < 0.01), suggesting that EV71 infection promotes activation of monocyte-derived iDCs (Figure 1B).

### Differential expression of molecules in MEK/ERK signaling pathway

At 1/2 h, 2 h, 8 h and 24 h p.i. with EV71, iDCs were collected and expression of molecules involved in the MEK/ERK signaling pathway was examined by PCR arrays. The results showed that the expression levels of KRAS, Raf-1, c-Fos, c-Jun, c-myc, SOS1, IL-1α, IL-2, IL-6, IL-12, TNF-α, IFN-β and IFN-γ mRNAs were upregulated by 2.04-16.7 folds in EV71-infected iDCs at different time points, while IFN-α1 expression was not changed within 24 h. Elk1 was downregulated by 2.57-fold at 1/2 h, but upregulated by 2.47-fold at 24 h p.i. However, EV71 infection did not enhance mRNA levels of MEK1/2 and ERK1/2 molecules (Table 1).

**Table 1:** **Differential expression of genes in MEK/ERK signaling pathway in EV71-infected iDCs at different time points**

**Gene symbol**	**EV71/control (Fold changes)**			
	**1/2 h**	**2 h**	**8 h**	**24 h**
KRAS	−1.09	+1.94	+2.21	+1.18
NRAS	−1.05	+1.08	+1.42	−1.07
Raf-1	+1.07	+6.51	−1.25	−1.21
MEK1	+1.26	+1.26	−1.02	−1.09
MEK2	+1.15	+1.19	−1.04	−1.15
ERK1	+1.27	−1.16	−1.10	−1.20
ERK2	+1.73	+1.23	−1.36	−1.65
c-Fos	+5.46	+7.65	+4.62	+3.71
c-Jun	+1.64	+2.05	+2.78	+3.67
c-myc	+1.20	−1.31	+2.54	−1.15
Elk1	−2.57	+1.94	+1.69	+2.47
SOS1	+1.89	+16.70	−1.78	−1.15
IL-1α	−1.00	+5.49	+3.82	−1.17
IL-2	+1.23	+3.12	+2.76	+1.79
IL-6	+1.68	+2.33	+2.84	+1.67
IL-12	+1.34	+1.76	+2.36	+1.84
IFN-α1	−1.56	+1.58	+1.37	−1.75
IFN-β	−1.26	+2.72	+2.04	−1.49
IFN-γ	−1.10	+2.69	+1.40	−1.82
TNF-α	+1.28	+2.27	+2.62	+1.41

Upregulated and downregulated transcripts are indicated as ‘+’ and ‘–’ values respectively. The values presented in the table are the average of three experiments.

### The effect of inhibitor U0126 on EV71 infection

**Figure 2:**
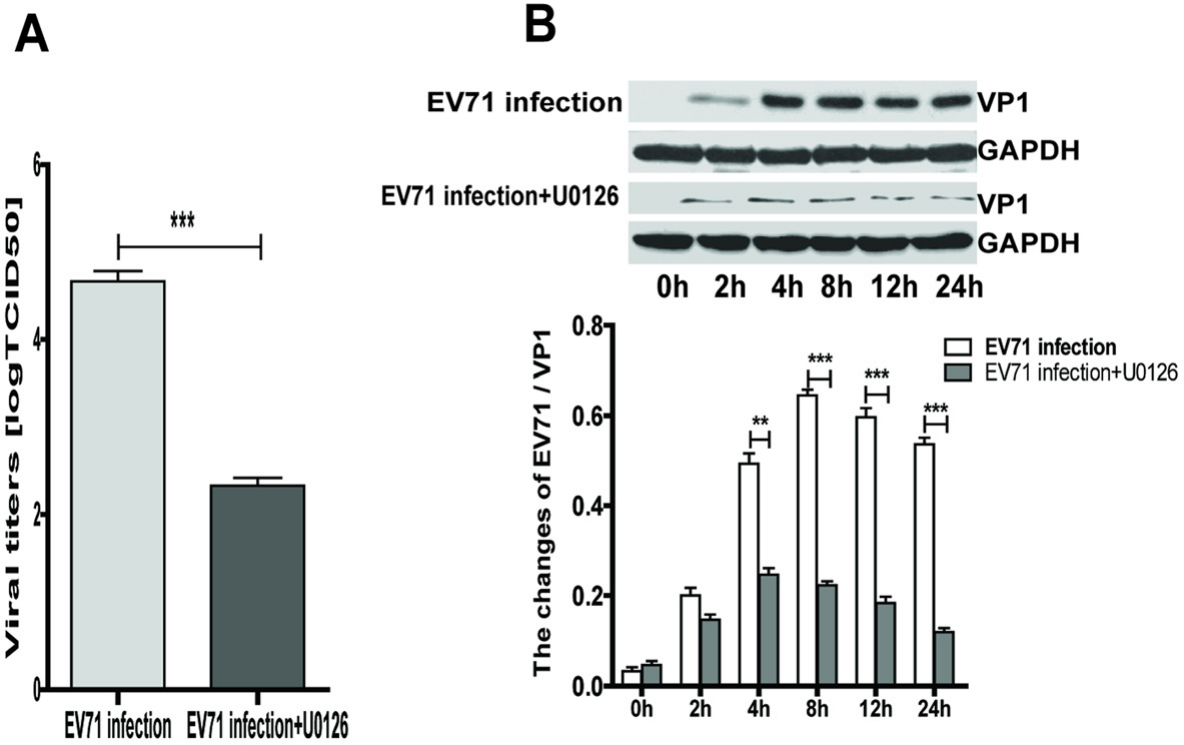
**The inhibitory effects of U0126 on EV71 replication. (A)** iDCs (3 × 10^5^/well) pretreated with or without U0126 (20 μM) for 1 h and infected with EV71 (MOI = 5) for 24 h. Culture supernatants were collected after infection to determine viral titers. **(B)** Western blot results of the supernatants and cell lysates of iDCs pre-incubated without or with U0126 (20 μM) for 1 h and infected with EV71 at a MOI of 5, using a specific antibody against VP1. The intensity of VP1 protein band was quantitated by densitometric analysis and normalized to GAPDH. The data were expressed as mean ± SE from three independent experiments and analyzed by two-way ANOVA with Bonferroni post-hoctests (**p < 0.01,***p < 0.001).

U0126, a specific inhibitor of MEK1/2, was used to study the effect of cellular ERK activation on expression of VP1, a capsid protein of EV71. Compared with EV71 infection, U0126 (20 μM) significantly reduced the EV71 virus titer as determined by TCID50 (Figure 2A). Meanwhile, the treatment with U0126 (20 μM) inhibited the expression of EV71/VP1 protein at 4 h, 8 h, 12 h and 24 h p.i., respectively (Figure 2B).

### EV71 infection triggered the activation of MEK1/2 and ERK1/2 molecules

**Figure 3:**
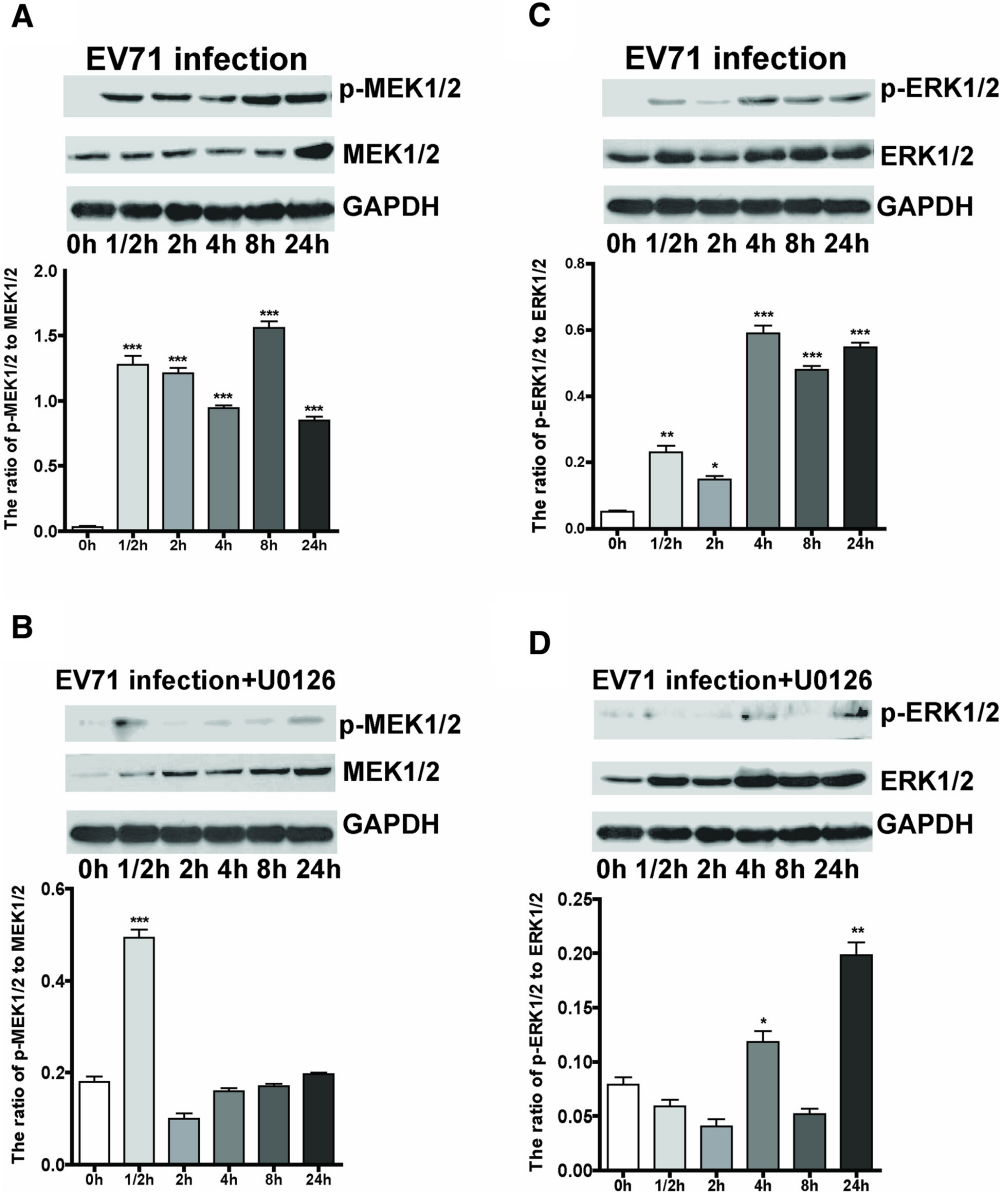
**Activation of MEK1/2 and ERK1/2 by EV71 infection and its inhibition by U0126.** iDCs were infected with EV71 at a MOI of 5. Cells were collected at 0 h, 1/2 h, 2 h, 4 h, 8 h and 24 h p.i., and lysed for western blot analysis. GAPDH was used as an internal control. The total or phosphorylated MEK1/2 and ERK1/2 at different time points were analyzed by western blot **(A and C)**. iDCs were pre-incubated with U0126 (20 μM) for 1 h, and then infected with EV71 at a MOI of 5, U0126 was present throughout the experiment **(B and D)**. The ratios of p-MEK1/2 to MEK1/2 and p-ERK1/2 to ERK1/2 at different times were analyzed by densitometric scanning. The data were expressed as mean ± SE from three independent experiments and analyzed by one-way ANOVA (*p < 0.05, **p < 0.01,***p < 0.001).

To determine whether the MEK/ERK signaling pathway is involved in EV71 infection, we examined the levels of total and phosphorylated MEK1/2 and ERK1/2 at 0 h, 1/2 h, 2 h, 4 h, 8 h and 24 h p.i. As shown in Table 1, the results showed that virus infection did not enhance mRNA levels of MEK1/2 and ERK1/2 molecules in PCR assay, but significantly elevated their phosphorylation (Figure 3A and C). Moreover, the enhanced phosphorylation was significantly suppressed by inhibitor U0126 (20 μM) in EV71-infected iDCs (Figure 3B and D). It demonstrates that the activation of Raf/MEK/ ERK kinase cascade plays an important role in EV71 replication cycle, and phosphorylation of ERK1/2 by MEK1/2 is a critical step in the MAPK cascade.

### EV71 infection stimulated the phosphorylation of transcription factor c-Fos, c-Jun, c-myc and Elk1

**Figure 4:**
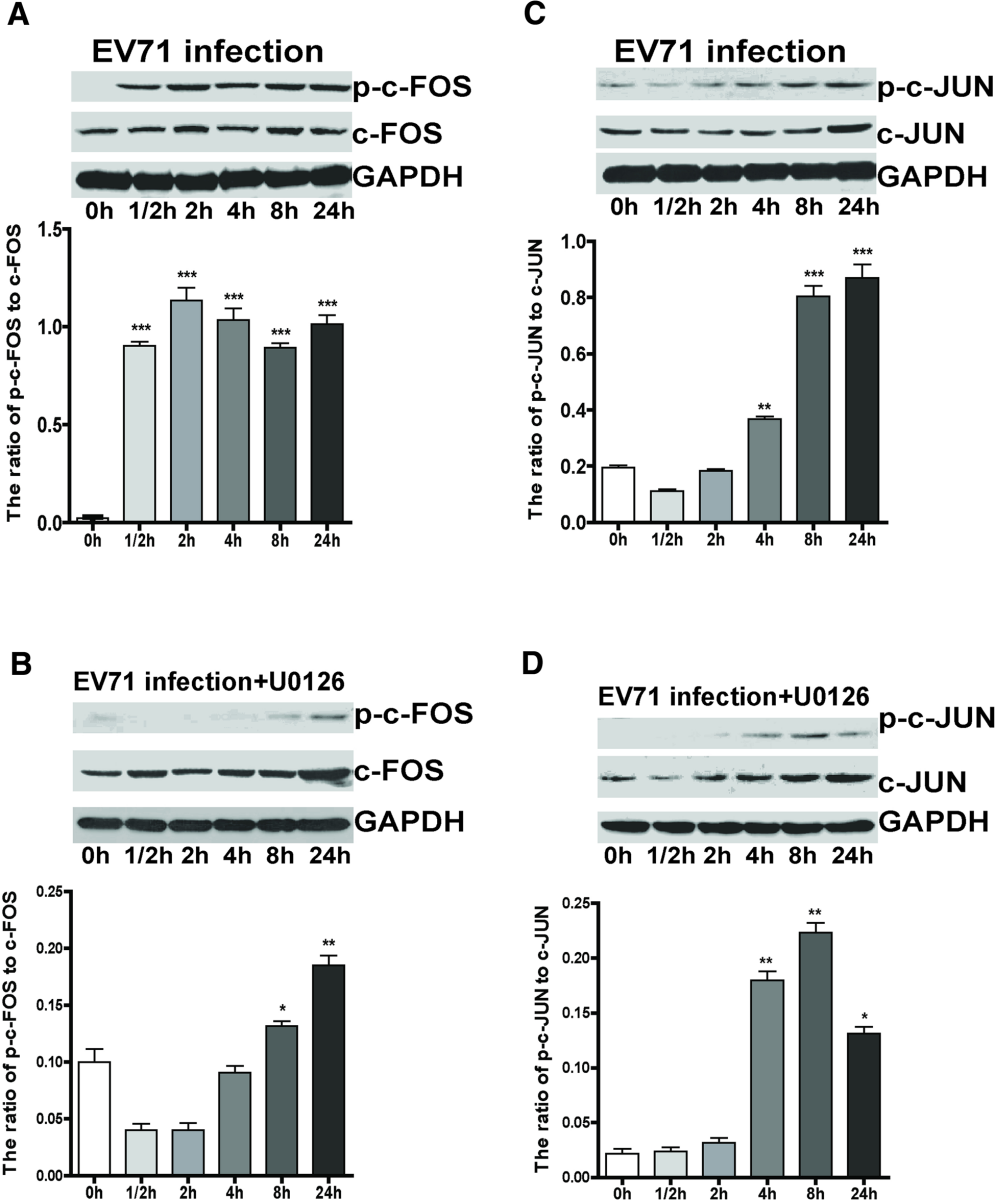
**Activation of c-Fos and c-Jun by EV71 infection.** iDCs were infected with EV71 at a MOI of 5. Cells were collected and lysed at 0 h, 1/2 h, 2 h, 4 h, 8 h and 24 h p.i., and GAPDH was used as an internal control. The total or phosphorylated c-Fos and c-Jun at different time points were analyzed by western blot **(A and C)**. iDCs were pre-incubated with U0126 (20 μM) for 1 h, and then infected with EV71 at an MOI of 5 **(B and D)**. The ratios of p-c-Fos to c-Fos and p-c-Jun to c-Jun at different times were analyzed by densitometric scanning. The data were expressed as mean ± SE from three independent experiments and analyzed by one-way ANOVA (*p < 0.05, **p < 0.01, ***p < 0.001).

**Figure 5:**
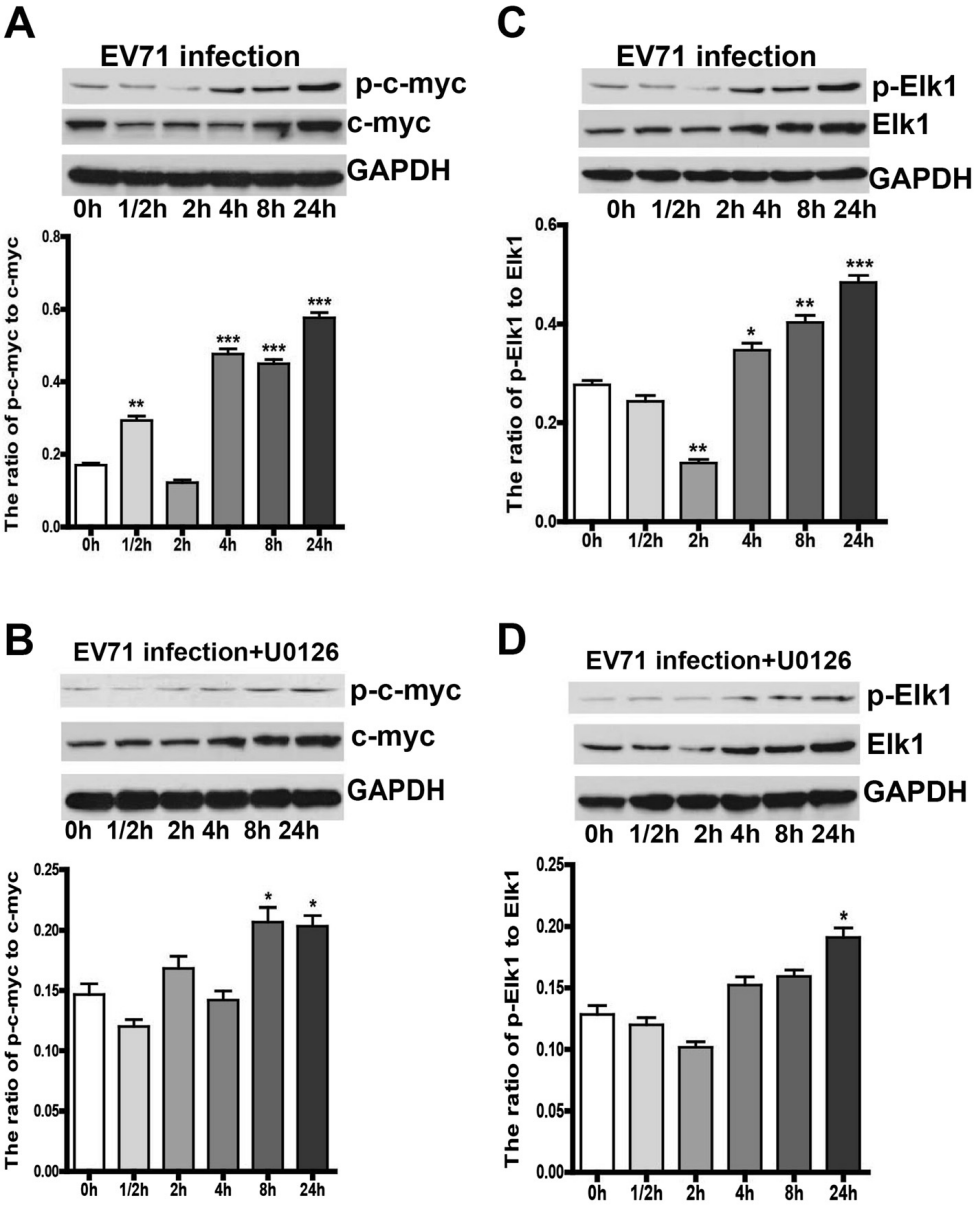
**The phosphorylation of nuclear transcription factor c-myc and Elk1. **iDCs were infected with EV71 at a MOI of 5. Cells were collected and lysed at 0 h, 1/2 h, 2 h, 4 h, 8 h and 24 h p.i., and GAPDH was used as an internal control. The total or phosphorylated c-myc and Elk1 at different time points were analyzed by western blot **(A and C)**. iDCs were pre-incubated with U0126 (20 μM) for 1 h, and then infected with EV71 at a MOI of 5 **(B and D)**. The ratios of p-c-myc to c-myc and p-Elk1 to Elk1 at different time were analyzed by densitometric scanning. The data were expressed as mean ± SE from three independent experiments and analyzed by one-way ANOVA (*p < 0.05, **p < 0.01, ***p < 0.001).

It is well known that phosphorylated ERK1/2 translocate from cytoplasm to the nucleus where they activate a variety of targets, including transcription factors c-Fos, c-Jun, c-myc and Elk1. Therefore, we examined the effect of EV71 infection on c-Fos and c-Jun, and found that the phosphorylation of c-Fos and c-Jun was enhanced in EV71-infected iDCs at a MOI of 5 (Figure 4A and C). Moreover, the enhancement was attenuated by treatment with U0126 (20 μM), 1 h prior to EV71 infection (Figure 4B and D). In addition, EV71 infection (MOI = 5) significantly increased the phosphorylation of Elk1 and c-myc, and the pretreatment with U0126 (20 μM) remarkably decreased their phosphorylation level (Figure 5). The data demonstrate that EV71 infection triggers ERK1/2-dependent activation of c-Fos, c-Jun, c-myc and Elk1.

### EV71 infection promoted the expression of SOS1 protein

**Figure 6:**
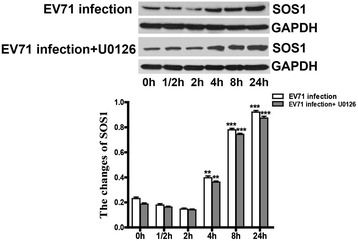
**EV71 infection stimulates the expression of SOS1 protein.** EV71 infection: EV71-infected iDCs. EV71 infection + U0126: EV71-infected iDCs in the presence of U0126 (20 μM). iDCs were infected with EV71 at a MOI of 5. Cells were collected and lysed at 0 h, 1/2 h, 2 h, 4 h, 8 h and 24 h p.i. SOS1 expression at different time points were analyzed by western blot. iDCs were pre-incubated with U0126 (20 μM) for 1 h, and then infected with EV71 at a MOI of 5. The intensity of SOS1 protein band was quantitated by densitometric analysis and normalized to GAPDH. The data were expressed as mean ± SE from three independent experiments and analyzed by one-way or two-way ANOVA (**p < 0.01, ***p < 0.001).

The SOS1, as an activator of Ras, which activates the MEK/ERK signaling pathway and plays a fundamental role in growth and cellular differentiation. As mentioned above, the expression of SOS1 gene was upregulated by 16.7-fold in EV71-infected iDCs at 2 h p.i. Compared with 0 h, 1/2 h and 2 h p.i., SOS1 protein level gradually increased at 4 h, 8 h and 24 h p.i., and no difference was observed in the presence of U0126 (20 μM) (Figure 6). Although the exact mechanism remains unclear between the activation of SOS1 and virus infection, it indicates that EV71 infection activates SOS1 gene, which may enhance the activation of Ras/Raf/MEK signaling cascade in EV71-infected iDCs.

### The secretion of cytokines and interferons in EV71-infected iDCs

**Figure 7:**
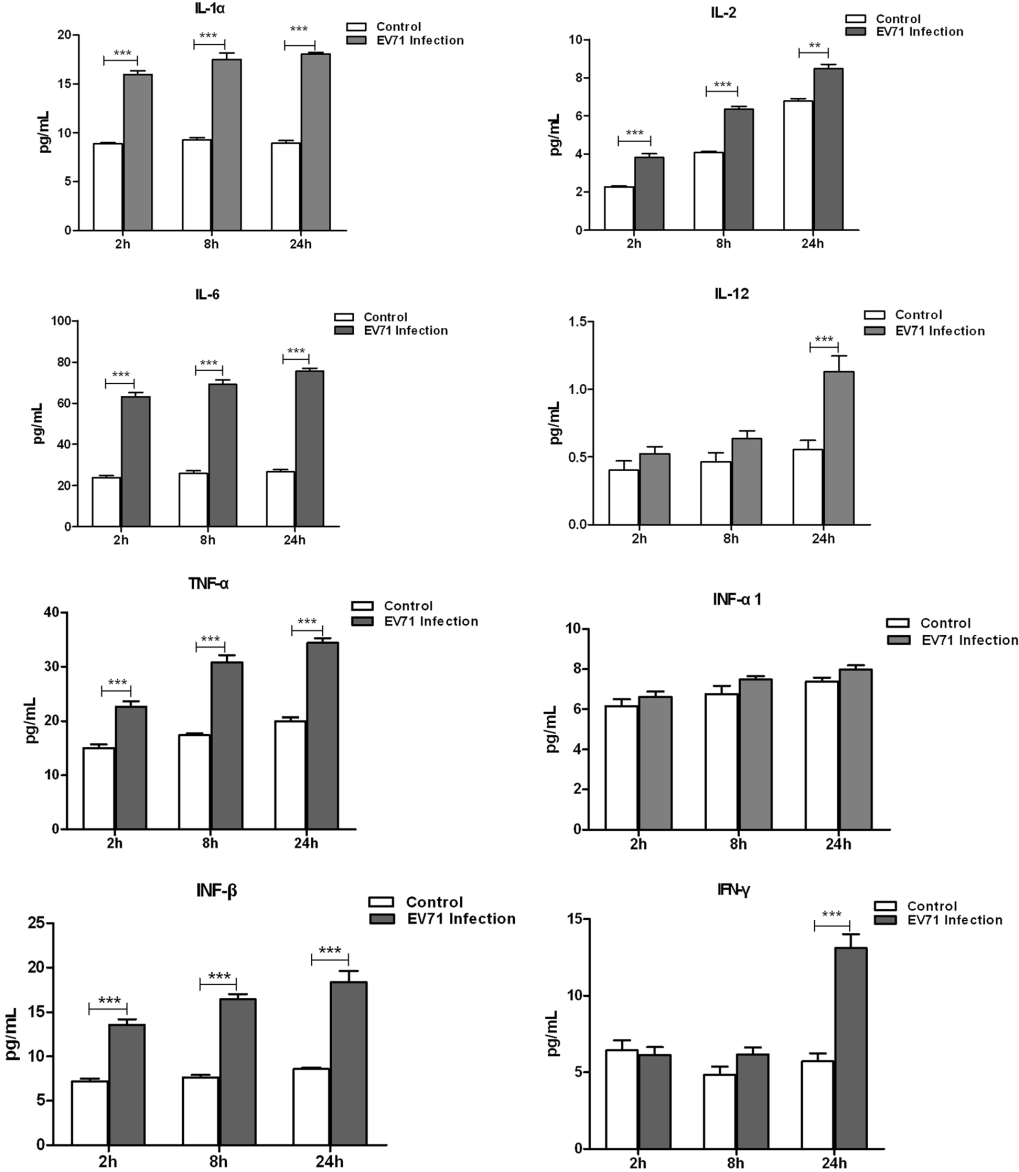
**EV71 infection promotes cytokine releases in iDCs.** The levels of cytokines in the culture supernatants of uninfected iDCs (control) and EV71-infected iDCs harvested at 2 h, 8 h and 24 h p.i. were measured by luminex fluorescence technique. The data were expressed as mean ± SE from three independent experiments and analyzed by two-way ANOVA with Bonferroni post-hoctests (**p < 0.01,***p < 0.001).

**Figure 8:**
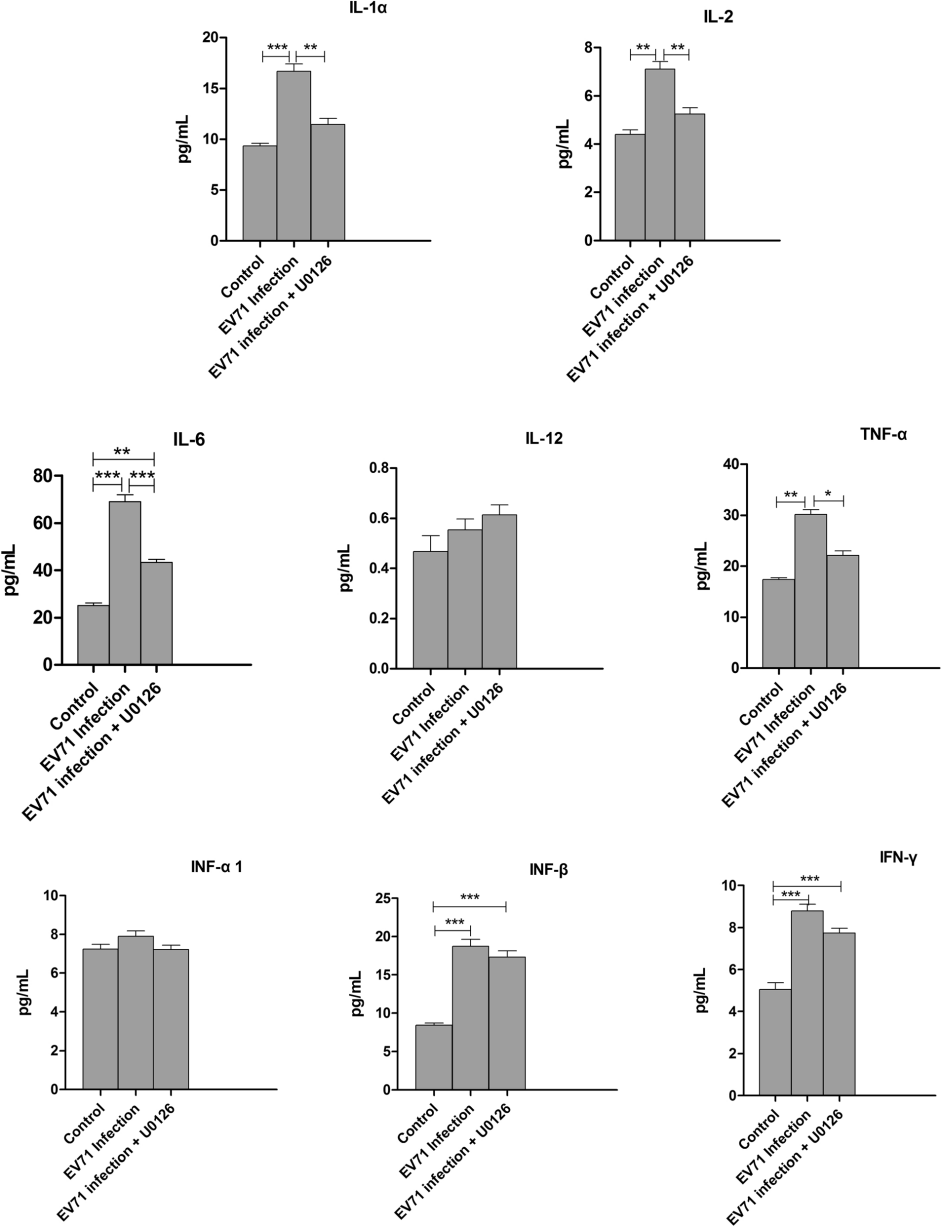
**U0126 inhibits cytokine releases in EV71-infected iDCs.** The culture supernatants of uninfected iDCs (Control), EV71-infected iDCs (EV71 infection) and U0126 treatment were harvested at 24 h p.i. and cytokines were detected by the luminex fluorescence technique. The data were expressed as mean ± SE from three independent experiments and analyzed by one-way ANOVA (*p < 0.05, **p < 0.01, ***p < 0.001).

DCs secrete cytokines and interferons once they are activated by bacterial or viral infection. Thus, we examined the levels of cytokines in culture supernatants collected at 24 h from control, EV71-infected iDCs and iDCs preincubated with U0126 (20 μM) using luminex fluorescent technique. The results showed that EV71 infection (MOI = 5) significantly increased secretions of IL-1α, IL-2, IL-6, IL-12, TNF-α,IFN-β and IFN-γ from iDCs (Figure 7). In addition, the secretions of IL-1α, IL-2, IL-6 and TNF-α were remarkably inhibited by U0126, whereas those of IL-12, IFN-α1, IFN-β and IFN-γ were not different between U0126 treated and EV71 infected cells (Figure 8).

## Discussion

DCs are essential for the induction of specific immune responses against invading pathogens [17,25]. Two distinct DC subsets were originally defined in human blood based on the expression of CD11c. The CD11c^+^ DCs are generally called myeloid DCs (mDCs) because they express myeloid markers, whereas CD11c^−^ DCs are called plasmacytoid DCs (pDCs) because of their plasma cell-like morphology and localization at the precursor stage [26,27]. Human DCs, especially iDCs, are highly specialized and efficient in uptaking and processing antigens, which have been shown to support the propagation of several viruses including EV71 and dengue viruses, and the infection of these viruses could increase the viability, activation, cytokine release and T-cell priming activity in DCs [28,29].

As previously reported, herpes simplex virus could infect both mature and immature DCs, whereas only iDCs support a productive infection, which could lead to apoptosis [30,31]. EV71 could also infect both mature and immature DCs, and replicate in them even though at slightly less and comparable amounts [28]. In this study, we isolated mononuclear cells from peripheral blood, purified monocytes and differentiated them into iDCs in the presence of GM-CSF plus IL-4. Subsequently, iDCs were infected by EV71 with a MOI of 5. Previous report showed that EV71 could activate iDCs and promote their T-cell priming activity, but failed to induce the maturation (CD83 expression) of iDCs [14]. However, we found that EV71 infection increased the percentages of CD83 and CD86 were expressed on iDCs. It might be related to viral titer, infection duration and experiment performance, and needed to be further studied. In addition, the percentages of CD80 and HLA-DR were not changed in EV71-infected iDCs. These results showed that EV71 infection could enhance the expression of maturation markers of iDCs and activate iDCs.

There are mainly three MAPK signaling pathways associated with virus infection, including ERK1/2, p38 MAPK, and JNK [32-35]. In contrast to the JNK cascade, activation of the Raf/MEK/ERK pathway in host cells (e.g., RD cells, HEK293 cells, vero cells, oval cells, and lymphocytes) seems to be required for virus replication, including EV71, herpes simplex virus 2, yellow fever virus, hepatitis B virus and human immunodeficiency virus type 1 [23,24,36-38]. Here, we showed that the gene expression levels of KRAS, Raf-1, c-Fos, c-Jun, c-myc, and Elk1, but not MEK1/2 and ERK1/2 in EV71-infected iDCs increased at 1/2 h, 2 h, 8 h and 24 h p.i., suggesting that EV71 infection could activate Ras and Raf-1, which in turn enhanced activation and phosphorylation of MEK1/2 and ERK1/2. Phosphorylated ERK1/2 subsequently translocated into nucleus, where they upregulated the transcription of c-Fos and c-Jun to promote EV71 replication. Furthermore, we found that the treatment with U0126 (20 μM) significantly reduced phosphorylation of MEK1/2 and ERK1/2, as well as their downstream molecules c-Fos, c-Jun, c-myc and Elk-1. Because the activation of these transcription factors participated in the regulation of cell proliferation and differentiation, we hypothesize that the activation of MEK/ERK signal cascade is required for EV71 replication in iDCs.

SOS was discovered in Drosophila melanogaster and plays an important role in normal eye development in Drosophila [39,40]. Human SOS (hSOS) has two homologues, SOS1 and SOS2, which are ubiquitously expressed in different human tissues and cell lines [41]. Upon growth factor stimulation, SOS1 is recruited by growth factor receptor-bound protein 2 (Grb2) to the plasma membrane with subsequent release of autoinhibition. Once SOS1 is activated, it interacts with Ras proteins to promote guanine nucleotide exchange (GDP/GTP) and subsequent formation of the active Ras-GTP complex which stimulates the activation of Ras/MEK/ERK sigaling pathway [42,43]. In this study, we found that EV71 infection significantly upregulated the expressions of SOS1 mRNA level and protein, which might further enhance the activation of Ras/MEK/ERK signaling pathway. Although the mechanism of SOS1 overexpression stimulated by EV71 infection is still unknown yet, we speculate that SOS1 plays an important role in EV71 infected-iDCs.

Several pro-inflammatory cytokines (e.g. IL-1, IL-6, TNF-α, and IFN-β, etc) are induced by oxidant stress, cytokines, and virus infection, which play a role in host cell damage, chronic inflammation, and other immunoresponses [44,45]. EV71 infection can induce iDCs to secrete various cytokines. PCR array showed that EV71 infection increased the expression of IL-1α, IL-2, IL-6, IL-12, TNF-α, IFN-β and IFN-γ, but not IFN-α1, by 2.04-5.49-fold in iDCs. In addition, EV71 infection stimulated the secretion of IL-1α, IL-2, IL-6, IL-12, TNF-α,IFN-β and IFN-γ as measured by luminex fluorescent technique. For these cytokines, a good correlation was obtained between PCR array and luminex data. Furthermore, treatment with ERK1/2 inhibitor U0126 (20 μM) decreased the production of IL-1α, IL-2, IL-6 and TNF-α through blocking the MEK/ERK signaling pathway.

## Conclusions

To our knowledge, this study is the first report to show the activation of MEK/ERK signaling pathway in EV71-infected iDCs. The results provide evidences that the activation of MEK/ERK pathway may be associated with EV71 replication in iDCs. Moreover, we found that inhibition of the MEK/ERK cascade could effectively reduce virus replication and lead to a diminished release of cytokines. The upregulation of SOS1 expression might further activate MEK/ERK signaling pathway. Together, these data demonstrate that MEK/ERK signaling pathway plays an important role in EV71-infected iDCs. Therefore, understanding the fundamental mechanisms of EV71-induced activation of MEK/ERK signaling pathway is important for development of new antiviral drugs.

## Materials and methods

### Ethics statement

The study was approved by the Ethics Committee of the Third Affiliated Hospital of Soochow University, and all patients provided informed consents.

### Antibodies and chemicals

Dulbecco's modified Eagle's medium (DMEM), fetal bovine serum (FBS) and RPMI 1640 were purchased from Thermo Scientific HyClone (UT, USA). Hybond C membrane and ECL Western blot detection system were from Pierce (Rockford, IL, USA). Rabbit polyclonal antibodies against MEK1/2, p-MEK1/2, ERK1/2, p-ERK1/2, c-Fos, p-c-Fos, c-Jun, p-c-Jun, p-c-myc, c-myc, Elk1, p-Elk1, and HRP conjugated goat anti-rabbit IgG were purchased from SAB (Pearland, TX, USA). SOS1 mouse monoclonal antibody was provided from abnova Company (Taiwan). Antibodies against anti-glyceraldehyde-3-phosphate dehydrogenase (GAPDH) were obtained from ProteinTECH Group (Chicago, IL, USA). Rabbit polyclonal antibody against EV71/VP1 was purchased from Abcam Company (Cambridge, UK). The MEK1/2 specific inhibitor U0126 was acquired from LC Laboratories (Woburn, MA, USA) and freshly prepared as DMSO solution.

### Cell culture and virus propagation

RD cells were cultured in high glucose DMEM (HyClone, USA) supplemented with 10% FBS at 37°C and in a humidified incubator with 5% CO_2_ atmosphere. When cells reached 90% confluence, the original media were removed and the monolayer cells were washed with PBS once. Approximately 1 × 10^6^ of RD cells were incubated with EV71 strain GDV083 (ATCC VR-784) at a MOI of 5 or as indicated and allowed for absorption for 1.5 h at 37°C. Infected cells and culture supernatants were collected at different time intervals. Viruses were propagated in up to 90% confluent cell monolayer in DMEM containing 2% FBS and antibiotics as described above. The viral titer was determined by cytopathic effects (CPE) and expressed as 50% tissue culture infective dose (TCID50) per ml [46].

### Generation of iDCs

PBMCs were isolated from healthy blood donors and purified by Ficoll-Hypaque (Invitrogen, CA, USA) density gradient centrifugation. Monocytes were isolated from PBMCs by adhesion to plastic dishes for more than 2 h at 37°C and subsequently cultured with GM-CSF (100 ng/ml) and IL-4 (50 ng/ml). iDCs were generated for 7 days and infected with EV71 at a MOI of 5 for 1 h at 37°C. After washed twice with PBS, iDCs were cultured in RPMI medium for 24 h, and unifected iDCs were used as control. The phenotypic patterns of CD3, CD11c, CD80, CD83, CD86 and HLA-DR were characterized by flow cytometry (Beckman coulter, CA, USA). Meanwhile, the supernatants were stored at -80°C.

### Total RNA preparation and PCR arrays

After incubating at 37°C for 1/2 h, 2 h, 8 h and 24 h, total cellular RNA was isolated from EV71-uninfected and infected iDCs using the Trizol reagent. Complementary DNA (cDNA) was generated from mRNA by reverse transcription with oligo (dT) primer. Then, 1 μg of cDNA was added into a 96-well plate pre-dispensed with the indicated primers (CT biosciences, China). PCR arrays were performed by LightCycler 480 (Roche Diagnostics, Germany). The thermocycler parameters were performed with an initial denaturation at 95°C for 5 min followed by 40 cycles of denaturation at 95°C for 15 s, annealing at 60°C for 15 s and extension at 72°C for 20 s. The housekeeping genes such as B2M, ACTB, GAPDH, RPL27, HPRT1 and OAZ1 were used to normalize RNA amount. Fold changes were calculated using the formula of 2^-ΔΔCt^. Each experiment was performed in triplicate.

### Cell extraction and western blot analysis

iDCs were pre-incubated for 1 h with U0126 at different concentrations as indicated and then infected with EV71 at a MOI of 5 in the presence of U0126 for 48 h. Cells were harvested by centrifugation, washed and lysed with 50 mM Tris pH 6.8, containing 250 mM NaCl, 2% sodium deoxycholate, 0.5% NP-40 and 1 mM PMSF. After vortexing and incubating on ice for 10 min, cell lysates were centrifuged at 10,000 × g for 5 min. Total protein concentration was determined by the bicinchoninic acid protein assay kit (Pierce). Proteins in the lysates were separated by sodium dodecyl sulfate polyacrylamide-polyacrylamide gel electrophoresis (SDS-PAGE). The separated proteins were transferred to PVDF membranes (Millipore), and probed with specific primary antibodies. After washed with PBS, the membranes were incubated with horseradish peroxidase conjugated secondary antibodies. The specific proteins were detected by ECL reagents (GE Healthcare), visualized on Super RX film (Fujifilm) and quantitated by densitometric analysis (ImageQuant, Molecular Dynamics and PDSI, GE Healthcare). The relative amounts of the phospho-ERK1/2 (p-ERK1/2) were normalized to GAPDH after densitometry scanning and analysis with the band leader software (version 3.0).

### Evaluation of cytokine levels by luminex fluorescent technique

iDCs were infected with EV71 at a MOI of 5 for 1 h at 37°C, washed twice and cultured in complete RPMI medium. At 8 h, 12 h, and 24 h p.i., the supernatants were collected by centrifugation (2,000 × g). As previously reported [47], the concentrations of IL-1α, IL-2, IL-6, IL-12, TNF-α, IFN-α1, IFN-β and IFN-γ in culture supernatant were analyzed with Milliplex Magnetic Beads (Millipore, USA) using luminex fluorescent technique.

### Statistic analysis

Quantitative data were presented as the mean ± SE and statistically analyzed using GraphPad Prism software (San Diego, CA). Qualitative data were analyzed with Chi-square test. A *p* value less than 0.05 was considered as being statistically significant.
